# The Ecology of Non-*Candida* Yeasts and Dimorphic Fungi in Cetaceans: From Pathogenicity to Environmental and Global Health Implications

**DOI:** 10.3390/jof10020111

**Published:** 2024-01-29

**Authors:** Victor Garcia-Bustos, Begoña Acosta-Hernández, Marta Dafne Cabañero-Navalón, Javier Pemán, Alba Cecilia Ruiz-Gaitán, Inmaculada Rosario Medina

**Affiliations:** 1 Universitary Institute of Animal Health and Food Security (ULPGC-IUSA), University of Las Palmas de Gran Canaria, 35416 Arucas, Spain; inmaculada.rosario@ulpgc.es; 2 Severe Infection Research Group, Health Research Institute La Fe, 46026 Valencia, Spain; marta.dafne.cabanyero@gmail.com (M.D.C.-N.); peman_jav@gva.es (J.P.); albacruiz@gmail.com (A.C.R.-G.)

**Keywords:** cetacean mycobiome, fungal infection, dolphin, one health, zoonosis, fungal pathogen

## Abstract

Cetaceans, which are integral to marine ecosystems, face escalating anthropogenic threats, including climate change and pollution, positioning them as critical sentinel species for ocean and human health. This review explores the neglected realm of non-*Candida* yeasts in cetaceans, addressing the gaps in the understanding of their prevalence, pathogenicity, and environmental impacts. By examining identified species such as *Cryptococcus* spp., *Paracoccidioides* spp., and several dimorphic fungi, this review emphasizes global prevalence, epidemiology and ecology, pathogenicity, and potential zoonotic implications. It also discusses the fine line between yeast commensalism and pathogenicity by considering environmental influences such as pollution, climate shifts, and immune suppression. Environmental impact discussions delve into how rising ocean temperatures and pollution can modify yeast mycobiota, potentially affecting marine host health and broader ecosystem dynamics. The cetacean’s unique physiology and ecological niches are considered, highlighting potential impacts on behaviors, reproductive success, and survival rates. Identifying crucial knowledge gaps, the review calls for intensified research efforts, employing advanced molecular techniques to unravel the cetacean mycobiome. Systematic studies on yeast diversity, antifungal susceptibility, and their influence on environmental and ecosystem health are proposed, and the balance between commensal and pathogenic species emphasizes the significance of the One Health approach. In conclusion, as marine mammals face unprecedented challenges, unveiling non-*Candida* yeasts in cetaceans emerges as a critical endeavor with far-reaching implications for the conservation of marine ecosystems and for both animal and human public health.

## 1. Introduction

Cetaceans play a critical role in marine ecosystems. Marine mammals are consumers of production at most trophic levels, and this position in the trophic hierarchy directly affects both predator and prey dynamics, thereby influencing marine biodiversity and nutrient cycling [1,2,3]. However, the environment is being increasingly affected by severe anthropogenic impacts such as climate change and pollution, among others. In this regard, cetaceans are considered sentinel species for both ocean and human health [4]. They are ideal indicators of ecosystem health due to their extended lifespan, enduring coastal residence, high-trophic level feeding, and distinct fat reserves that accumulate anthropogenic toxins. As many marine mammals inhabit coastal regions alongside humans and share dietary sources, they can also act as reliable indicators of potential human health concerns [5]. In fact, they face numerous environmental challenges, such as chemical pollution, temperature and salinity fluctuations, and algal toxins. Importantly, they play a significant role in the emergence and spread of both new and re-emerging pathogens [6].

Non-*Candida* yeasts might play a conspicuous role in the complex microbiota ecology of cetaceans [7]. Current research, despite being considerably limited, points to the considerable biodiversity of these yeasts within different cetacean hosts, and there is still a significant knowledge gap on the role of these microorganisms in the ecology and pathology of their hosts. Although several studies on bacterial microbiota have been conducted in cetacean populations, the fungal microbiome and fungal-related pathologies have been largely ignored [8,9,10,11]. Notably, the distribution of these yeasts is not homogeneous and demonstrates significant intraspecies variation, as well as distinct anatomical niches within the same individual, including the skin, blowhole, and gastrointestinal tract, among others. These findings underscore the intricate interplay of host-specific and location-specific factors in shaping the composition of the fungal community. Several genera and species have been identified in both wild and captive cetacean populations. However, efforts in research have neglected fungal pathology and the mycobiome; therefore, the related evidence is considerably limited.

Therefore, this review aims to provide in-depth insight into the current evidence on non-*Candida* spp. yeasts in cetaceans, with an emphasis on prevalence, pathogenicity, clinical manifestations, and species distribution. We endeavor to collate findings from both captive and wild cetaceans and emphasize their relationship with human and environmental health from a One Health perspective. Furthermore, we address the main knowledge gaps and pinpoint areas for further research.

## 2. Identified Species of Non-*Candida* Yeasts in Cetaceans

### 2.1. Cryptococcus spp.

Cryptococcosis, caused by the fungi *Cryptococcus neoformans* or *C. gattii*, is increasingly significant in healthcare. *Cryptococcus neoformans* primarily affects immunocompromised individuals, often leading to central nervous system (CNS) complications and subsequent meningoencephalitis. In contrast, *C. gattii* infection typically results in severe lung disease. Globally, cryptococcosis sporadically impacts a broad range of species, from Acanthamoeba to large mammals [12,13]. While domesticated animals are well documented in the literature, free-living wildlife is often overlooked. The disease’s prevalence varies and is notably higher in parts of Australia, Brazil, and the Pacific Northwest. This fungal infection affects diverse wildlife, pets, livestock, and humans and can cause both overt disease and subclinical infection [14].

Cetacean species exhibit enhanced susceptibility to *Cryptococcus* spp. infections. Both *C. neoformans* and *C. gattii* have been identified in these animals (Table 1). While *C. neoformans* is ubiquitously distributed and frequently found in environments with minimal exposure to solar radiation, with a significant reservoir being shielded avian guano accumulations, *C. gattii* has a more restricted distribution and is commonly linked to the Eucalyptus camaldulensis tree, among others [14,15].

Unlike other fungal species, *Cryptococcus* spp. is not believed to be a normal colonizing organism in cetaceans, and mainly all reported cases are related to invasive diseases with high mortality rates [16]. This vulnerability is particularly pronounced during coastal migrations, while in captivity or in situations of proximity to terrestrial regions that allow the individuals to encounter infectious propagules present in effluents and runoff entering marine environments [14]. Additionally, these infections can occur as outbreaks and can be associated with the detection of cases in humans or other animal species [17]. In fact, previous cryptococcal epizootics that cause remarkable mortality in odontocetes are clustered around terrestrial hotspots [16]. The emergence of *C. gattii* in North America in 1999 marked a multispecies cryptococcosis outbreak across British Columbia, Washington State, and Oregon. Since the early 2000s, the Pacific Northwest of the USA and Canada, witnessed an upsurge in such infections among marine mammals and humans. Significantly, animal cases outnumbered human cases by approximately 75%, with a substantial impact on marine mammals [16,17,18].

The primary mode of transmission in cetaceans is through the inhalation of basidiospores. This susceptibility arises from the large intake of these infective propagules, which are transported deep into the lower respiratory system as a consequence of the inspiration of a large tidal volume after explosive exhalations, as well as the absence of sinonasal filtration mechanisms [14]. Furthermore, given the anatomical absence of a cribriform plate in cetaceans, direct inoculation and consequent neurological diseases are less frequent than in humans, in whom the prevalence of meningoencephalitis is considerably greater [19]. Pneumonia is the predominant clinical manifestation in cetaceans and can potentially escalate to disseminated systemic infections. It appears in the context of direct fungal invasion of the lung parenchyma and destructive inflammatory infiltration, sometimes associated with granulomatosis [20], bronchitis, and pleuritis [21]. It is usually followed by generalized lymphadenopathies and, in some cases, multiorgan affectation with gastric, renal, splenic, or even adrenal involvement [22,23]. A unique case of maternal–fetal transmission of *C. gattii* in a harbor porpoise (*Phocoena phocoena*) was also reported [24]. While some cutaneous lesions have been described [22,25], nearly all cases in the literature report pulmonary cryptococcosis.

Cryptococcal invasive disease can occur both in wild and captive animals [23,26]. On the one hand, *C. neoformans* infections have been reported in baleen whales such as the southern right whale (*Eubalaena australis*) coinfected with *Candida zeylanoides*. However, the majority of related studies have focused on odontocetes, specifically Dall’s porpoises (*Phocoenoides dalli*) and harbor porpoises. These investigations primarily involve necropsies conducted on wild stranded animals [17,25,26,27,28]. On the other hand, *C. gatti* infections have affected bottlenose dolphins (*Tursiops truncatus*), spinner dolphins (*Stenella longirostris*), Dall’s and harbor porpoises, and Pacific white-sided dolphins (*Lagenorhynchus obliquidens*) [16,17,18,21,22,24]. These isolations have been reported worldwide, in the West and East Atlantic, East Pacific, and Indian Ocean, and from the outbreaks of British Columbia to South Africa or Western Australia (Table 1).

Although *Cryptococcus* species are not typically considered standard colonizer in these species and are frequently associated with invasive cases during outbreaks, findings from two distinct studies employing both culture-based and molecular methodologies could challenge this understanding [29,30]. In 1990, an investigation into the microbiota obtained from cultures of healthy and lesional skin tissue samples from 19 bowhead whales (*Balaena mysticetus*) near Barrow, Alaska, identified two isolates of *C. neoformans* in lesioned skin. Additionally, several other species, including *C. gastricus*, *C. luteolus*, *C. albidus*, *C. laurentii*, *C. terreus*, and *C. uniguttulatus,* were detected in both healthy and lesional skin samples [29]. In a more recent study examining the gastrointestinal microbiota of East Asian finless porpoises (*Neophocaena asiaeorientalis sunameri*) using high-throughput sequencing, *Cryptococcus* spp. was very frequently detected in stomach, hindgut, and fecal samples. This was in stark contrast to the findings of the fecal microbiota of Californian blue whales (*Balaenoptera musculus*), where *Metschnikowia* spp. predominated.

While the strains of the fungus detected across various hosts have shown temporal and spatial consistency in known epizootics, the literature on this topic is fragmented. The validity of these studies is often contingent upon the diagnostic methods used. A notable research gap exists in antifungal susceptibility, with limited data available, as exemplified by a single report on itraconazole susceptibility in a bottlenose dolphin with cryptococcal bronchopneumonia [21]. This indicates a crucial area for further investigation to enhance the understanding and treatment of fungal diseases.

Moreover, the role of human activities, such as construction and deforestation, in the epidemiology of cryptococcal disease across species, including cetaceans, other animals, and humans, in environmental alterations demands attention. These activities can disturb habitats and facilitate the aerosolization of fungal spores, potentially contributing to disease proliferation, as observed in *C. gattii* outbreaks [16]. This connection accentuates the need for an interdisciplinary approach, integrating environmental, animal, and human health considerations to address the complex interplay between anthropogenic activities and disease dynamics, ultimately fostering a healthier coexistence between humans and their environment.

### 2.2. Paracoccidioides ceti: The Etiologic Agent of Lacaziosis or Lobomycosis

Paracoccidioidomycosis, previously known as lacaziosis or lobomycosis, was first documented by Jorge de Lobo in 1931 in a man from the Amazonia who presented with enduring sacral nodular lesions [31]. By 1971, a similar condition was observed in dolphins [32]. Given their phenotypic parallelism and cultivation challenges, both of these conditions were believed to be caused by a shared fungal agent, referred to as *Lacazia loboi*, by Taborda in 1999. Molecular studies subsequently revealed that the uncultivable pathogens causing this disease in dolphins and humans were distinct species: *P. ceti* and *P. lobogeorgii*, respectively [33,34]. However, *P. ceti* is assumed to be a zoonotic pathogen, as shown by recorded cases of human–dolphin transmission [35]. While such cases exist, they appear infrequently [36,37]. Unintended transmissions in research settings [38] and deliberate experimental infections in animals and humans have been documented [39,40].

Although human cases occur mainly in the rainforest regions of Central and South America, especially in the Brazilian Amazon basin [41], most dolphin infections have been reported along Florida’s coastline [42]. Nevertheless, there are accounts from distant areas, encompassing the Eastern and Western Atlantic, Eastern and Western Pacific, and Indian Ocean. It has been recognized in various locations in the Americas, including Brazil [43,44], Costa Rica [45], Venezuela [46], and Surinam [47]. Importantly, outside of the Americas, paracoccidioidomycosis has been reported in France [35], Spain [48], Madagascar [49], South Africa [50], and Japan [51,52] (Table 1). Nonetheless, all human cases in nonendemic countries have been imported.

To date, all cases of paracoccidioidomycosis have occurred in species of the *Delphinidae* family, while there are no reports on this phenomenon in platanistoid dolphins or mysticetes. Moreover, both captive and wild dolphins are susceptible to this infection, and serological studies have demonstrated that the seroprevalence against *P. ceti* in captive dolphins is 61.0%, while that in wild Dall’s porpoises is 26.9% [53]. Evidence of paracoccidioidomycosis has been reported in bottlenose dolphins (*T. truncatus*) in the West Atlantic [43,46,54,55,56], in the East Atlantic [35], and in the Eastern Pacific [45,57], and in Indo-Pacific bottlenose dolphins (*Tursiops aduncus*), both in the Indian Ocean [49] and in the Western Pacific [52,58]. Furthermore, there have been cases in Indian Ocean humpback dolphins (*Sousa plumbea*), in Australian snubfin dolphins (*Orcaella heinsohni*), and in Guiana dolphins (*Sotalia guianensis*) in the Western Atlantic [50,59] (Table 1).

The disease manifests through distinct clinical features, and it is often influenced by environmental factors, especially in epizootic instances observed in the coastal regions of Florida and North Carolina [60]. Its typical lesions, white to reddish and occasionally gray, are raised and adopt a nodular or verrucous profile, resembling the appearance of a cauliflower. They might ulcerate or become expansive plaques prone to bleeding upon minor trauma. Commonly impacted anatomical areas include the dorsal cranial surface, anterior dorsum, and fins [42,51,56,58,60,61]. Pathological features include acanthosis, hyperkeratosis, hyperpigmentation, profound fibrosis [42,52,58], lymphohystiocytic infiltration, and microabscesses replete with yeast-like cells connected by short and thin isthmuses [60].

However, many of the reports in the literature describing this disease involve only phenotypic characterizations of the lesions, both in wild and captive environments, and lack a proper microbiological identification of the etiological agents. Therefore, results must be carefully interpreted, especially considering the taxonomical chaos that governs the definition of the disease and considering that other fungal pathogens may be the cause of similar cutaneous diseases, such as *Trichosporon* spp. [62,63]. In fact, in many previous reports, lesions have been characterized as lobomycosis-like disease or lacaziosis-like disease—or, currently, paracoccidioidomycosis-like disease—when the histological or molecular detection of the pathogen has not been feasible [43,49,57,63].

**Table 1:** *Cryptococcus* spp. and *Paracoccidioides ceti* in cetaceans. Colonization was considered when there was no attributable evidence of infection or fungus-associated lesions reported.

Fungal Species	Cetacean Species	Colonization or Infection	Location	Isolation Origin	Captivity of Free-Living	Antifungal Resistance	Reference
Non-identified *Cryptococcus* spp.	*Neophocaena asiaeorientalis sunameri* *Stenella coeruleoalba, Tursiops truncatus*	Infection or colonization	China Western Australia	Lung, lymph nodes, stomach	Captive and free-living	No data	[23,26,30]
*C. neoformans*	*Eubalaena australis*, *Balaena mysticetus*. *Phocoena phocoena*, *Phocoenoides dalli*	Infection	Alaska British Columbia South Africa	Skin, lung, lymph nodes	Free-living	No data	[17,25,27,29]
*C. gattii*—VGI and VGIIa	*Lagenorhynchus obliquidens*, *Stenella longirostris*, *T. truncatus*, *P. dalli*, *P. phocoena*	Infection	Atlantic coast of Canada British Columbia California Hawaii South Africa Washington	Skin, lung, lymph nodes, stomach, adrenal gland, kidney, spleen, pleura, placenta, brain and meninges	Captive and free-living	One isolate in *T. truncatus* susceptible to itraconazole	[16,17,18,21,22,24]
*C. albidus*	*B.mysticetus*	Probable infection	Alaska	Skin	Free-living	No data	[29]
*C. gastricus*	*B.mysticetus*	Probable infection	Alaska	Skin	Free-living	No data	[29]
*C. luteolus*	*B.mysticetus*	Probable infection	Alaska	Skin	Free-living	No data	[29]
*C. laurentii*	*B. mysticetus*	Colonization	Alaska	Skin	Free-living	No data	[29]
*C. terreus*	*B.mysticetus*	Colonization	Alaska	Skin	Free-living	No data	[29]
*C. uniguttulatus*	*B.mysticetus*	Colonization	Alaska	Skin	Free-living	No data	[29]
*Paracoccidioides ceti*	*Orcaella heinsohni, P. dalli*, *Sotalia guianensis*, *Sousa plumbea, Stenella frontalis*, *T. truncatus*, *Tursiops aduncus*	Infection	Australia Belize Brazil Colombia Costa Rica Ecuador Florida Japan Madagascar Mayotte Mexico Peru South Africa Surinam Venezuela	Skin	Captive and free-living	No data	[35,42,43,46,48,49,51,52,57,61,63]

### 2.3. Other Dimorphic Fungi

In addition to *P. ceti*, other dimorphic fungi responsible for endemic systemic mycoses in other animals and humans have been reported in cetaceans, as seen in Table 2. In this section, we will review the current evidence for *Blastomyces* spp., *Coccidioides* spp., and *Histoplasma* spp. in these marine mammals.

#### 2.3.1. *Blastomyces* spp.

Blastomycosis, caused by the dimorphic fungus *Blastomyces dermatitidis*, is a prevalent disease in various species, including humans. Contracted primarily through the inhalation of airborne conidia, it manifests mainly in the lungs but can disseminate systemically. The eastern regions of the United States, especially around the Mississippi and Ohio River Basins, are noted hotspots [64].

A significant case of an Atlantic bottlenose dolphin from the Gulf of Mexico exhibited severe symptoms, initiating with an abscessed lesion in the melon and subsequent invasive disease [65]. Necropsy revealed extensive yeast cell invasion in the lung parenchyma and renal structures. Thoracic lymph nodes exhibited severe necrosis and yeast infiltration. Fungal cells across all affected organs, including the heart, liver, spleen, and gastrointestinal tract, were consistent with *B. dermatitidis*, as shown by specific immunofluorescence of the tissue and detection of specific serum precipitins by immunodiffusion tests. Interestingly, a treating veterinarian also contracted cutaneous blastomycosis, with a positive culture against *B. dermatitidis*, emphasizing the zoonotic potential of the pathogen. A systemic mycosis study in marine mammals reported a dolphin with blastomycosis, yet details on lesion location and severity were omitted [66].

#### 2.3.2. *Coccidioides* spp.

Coccidioidomycosis, caused by *Coccidioides immitis* or *Coccidioides posadasi*, is a zoonotic and highly pathogenic fungal infection endemic to the American continent. The fungus thrives in soil but is also resilient in saline environments, such as seawater. It releases arthroconidia, which, upon inhalation, can cause coccidioidomycosis in humans and animals [67].

The geographical distribution of *Coccidioides* spp. is experiencing an expansion, with new cases being identified in areas previously not recognized as endemic, such as eastern Washington, Oregon, and Utah. This expansion is largely attributed to a combination of climatic changes and human activities that disrupt the soil, such as military maneuvers, recreational activities, agriculture, and construction. These disturbances lead to the dispersion of arthroconidia, which become airborne and can be inhaled. The increased incidence of infection is also thought to be influenced by population changes. While most infections are asymptomatic, the disease can lead to severe infections in humans. These factors underline the growing concern for both marine wildlife and human populations in these expanding endemic regions [68].

A case in 1995 revealed a wild adult female bottlenose dolphin in La Jolla, California, infected with *C. immitis*, which developed dyspnea and rapid clinical deterioration until death with a clinical diagnosis of pneumonia [69]. Histological examination highlighted the presence of *C. immitis* in the lungs, lymph nodes, and brain. DNA testing and serology further confirmed the etiology and diagnosis of disseminated coccidioidomycosis. While infections have been previously documented in species such as California sea lions and sea otters [20], this was the inaugural finding in a free-ranging purely aquatic marine species and confirmed the fungus thrived in the marine environment, contrary to the usual arid or semiarid endemic zones. A more recent study by Kanegae et al. [53] revealed a 15.4% seroprevalence against *C. posadasii* in porpoises stranded in Hokkaido, Japan, with positivity in four Dall’s porpoises and one harbor porpoise. Taken together, these findings indicate an expanded environmental and host range for *Coccidioides*, emphasizing its adaptability and potential risks to marine wildlife. Further research is crucial to determining the comprehensive epidemiology of this fungus in marine species.

#### 2.3.3. *Histoplasma* spp.

*Histoplasma capsulatum*, a dimorphic fungus, is the etiological agent of disseminated histoplasmosis, and it is notably prevalent in individuals with compromised cellular immunity. The fungus is distributed globally, with notable endemicity in the Mississippi and Ohio River valleys of North America and select locales in Central and South America. Its prevalence is closely tied to soil disturbances, particularly in areas enriched with birds or bat guano. Notably, environmental changes, including climate alterations and anthropogenic land modifications, are impacting habitats conducive to *H. capsulatum* proliferation. These changes, in turn, influence disease epidemiology, highlighting the direct connection between environmental health and disease dynamics [70].

This pathogen has been described in Atlantic bottlenose dolphins in California, for which the techniques used ranged from fungal culture to PCR [27,71]. In one case, a 37-year-old female dolphin died following a five-month history of disseminated histoplasmosis, and the infection was confirmed by culture, PCR, and histopathology. Another case involved a 20-year-old male dolphin diagnosed with disseminated histoplasmosis and, at the same time, serum antigen levels. Interestingly, retrospective serum assays in both animals revealed longstanding elevated antigen levels for more than 20 years, even in the previous absence of severe invasive disease [71]. Moreover, a comprehensive 30-year retrospective assessment centered on pneumonia in bottlenose dolphins from the U.S. Navy Marine Mammal Program revealed that half of the 42 dolphins evaluated manifested pneumonia, as confirmed by histopathology. Among these cases, a 35-year-old female was diagnosed with a disseminated fungal infection attributed to *H. capsulatum* [27]. As with *Coccidioides* spp., the persistence of these organisms in marine settings has largely not been explored. However, animals might be in contact with the pathogen when they reach coastal regions, develop a latent infection, and ultimately invade disease as a consequence of immune disruption due to aging, pollution, or, in the case of captive individuals, immunosuppressive therapies such as glucocorticoids [71].

**Table 2:** Dimorphic fungi in cetaceans.

Fungal Species	Cetacean Species	Colonization or Infection	Location	Isolation Origin	Captivity of Free-Living	Antifungal Resistance	Reference
*Blastomyces dermatitidis*	*Tursiops truncatus*	Infection	Gulf of Mexico	Skin, lung, kidney, lymph nodes, heart, spleen, liver, gastrointestinal tract,	Free-living	No data	[65]
*Coccidioides immitis*	*T. truncatus*	Infection	California	Lung, lymph nodes, brain	Free-living	No data	[69]
*Coccidioides posadasii*	*Phocoena phocoena*, *Phocoenoides dalli*	Unknown	Japan	Serological evidence	Free-living	No data	[53]
*Histoplasma capsulatum*	*T. truncatus*	Infection	California	Lung	Captive	No data	[27,71]

#### 2.3.4. *Trichosporon* spp.

Yeasts of the genus *Trichosporon* spp. commonly cause superficial mycoses and are among the leading basidiomycetous yeasts causing invasive infections in humans. The clinical manifestation of *Trichosporon* infections depends on the site affected, with most invasive cases presenting with fungemia. Treatment options for *Trichosporon* spp. are limited, with azoles being the primary choice because of the inherent resistance of these yeasts to echinocandins [72].

*Trichosporon* spp. have been previously isolated from cetaceans (Table 3), either from healthy individuals or as a cause of cutaneous disease, which prompts the phenotypical differential diagnosis of paracoccidioidomycosis [29,62,73,74]. *Trichosporon asteroides* was cultured from multiple uneven skin lesions on a female bottlenose dolphin caught off the Japanese coast and housed in an outdoor aquarium in Japan, with clinical suspicion of paracoccidioidomycosis. However, the clinical characteristics of the lesions with multiple protuberances on the skin and the absence of typical cauliflower-like lesions were not characteristic [62].

In a previous study by Shotts and colleagues on bowhead whales in Alaska [29], *T. beigelii* was isolated twice from both lesional skin tissue and healthy skin samples, which highlights the possibility that *Trichosporon* species may act as both saprophyte colonizers and infective agents in cetacean skin. These findings are in line with those of other reports, such as those of Buck et al. [74], in which *T. cutaneum* (currently transferred to the genus *Cutaneotrichosporon*) was isolated from blowhole and fecal samples from bottlenose dolphins in Florida. Furthermore, again, in *T. truncatus*, Morris et al. [73] isolated *T. beigelii* in gastric, fecal, and blowhole samples from healthy wild individuals captured on the southeastern Atlantic coast of the United States in the absence of cutaneous manifestations. However, a key limitation in these studies is the absence of samples from non-lesional skin. These samples could provide insights into whether *Trichosporon* spp. are mere transient residents or stably colonize cetacean skin without inducing pathology. While *Trichosporon* yeasts are undeniably crucial players in human fungal infections, their exact role in cetaceans has yet to be elucidated. We believe that a more nuanced understanding, involving extensive sampling and comprehensive diagnostic approaches, is required to discern the true nature of the interaction of these organisms with marine mammals.

#### 2.3.5. *Rhodotorula* spp.

*Rhodotorula* spp. is a genus of pigmented yeasts known for their distinctive pink-to-coral appearance in culture. While most considered a benign environmental yeast found in various habitats, including soil, water, and air, *Rhodotorula* spp. Have been recognized as opportunistic pathogens in both humans and animals [75,76].

To date, there is no evidence of invasive infections caused by *Rhodotorula* spp. In cetaceans (Table 3). In Alaskan bowhead whales, isolates of *R. glutinis* have been predominantly identified from skin lesions, although they have also been detected on healthy skin. Conversely, *R. mucilaginosa* and *R. minuta*—now referred to as *Cystobasidium minutum*—have been exclusively associated with skin lesions [29]. Nevertheless, the clinical implications and potential pathogenicity of these isolations remain undetermined. According to a study by Buck [77], species such as *R. mucilaginosa*, *R. glutinis*, *R. graminis*, and *R. minuta* were found in the tanks housing captive bottlenose dolphins in Connecticut; however, no isolates were directly obtained from the dolphins within the facility.

#### 2.3.6. Other Yeasts

The genus *Malassezia* comprises lipophilic yeasts that act as both skin commensals and opportunistic pathogens in animals, including humans. *M. pachydermatis* is linked to otitis externa and various forms of dermatitis in dogs and cats [78]. While this pathogen has been associated with cutaneous disease in marine mammals such as pinnipeds [79,80], there is no evidence of *Malassezia*-associated disease in purely aquatic animals such as cetaceans. However, in a study using high-throughput sequencing of the fungal community at the genus level in the gastrointestinal tract of East Asian finless porpoises, *Malassezia* spp. were highly abundant in the foregut [30] (Table 3).

Using a similar culture-independent approach, Guass et al. [81] reported that *Metschnikowia* spp. were the dominant fungal species in two wild blue whales on the coast of California.

Finally, *Saccharomyces cerevisiae*, which plays a crucial role in the food industry, biotechnology, scientific research, and human health, was cultured from both the lesioned and healthy skin of Alaskan bowhead whales [29]. The significance of these isolations is uncertain.

**Table 3:** Other non-*Candida* yeasts in cetaceans.

Fungal Species	Cetacean Species	Colonization or Infection	Location	Isolation Origin	Captivity of Free-Living	Antifungal Resistance	Reference
*Trichosporon asteroides*	*Tursiops truncatus*	Infection	Japan	Skin	Free-living	No data	[62]
*T. beigelii*	*Balaena mysticetus*	Colonization and infection	Alaska	Skin	Free-living	No data	[29,74]
*Cutaneotrichosporon cutaneum*	*T. truncatus*	Colonization	Florida, South Carolina	Blowhole and faeces	Free-living	No data	[73]
*Rhodotorula glutinis*	*B. mysticetus*	Colonization or infection	Alaska	Skin	Free-living	No data	[29]
*R. mucilaginosa*	*B. mysticetus*	Colonization or infection	Alaska	Skin	Free-living	No data	[29]
*R. minuta -Cystobasidium minutum*	*B. mysticetus*	Colonization or infection	Alaska	Skin	Free-living	No data	[29]
*Malassezia* spp.	*Neophocaena asiaeorientalis sunameri*	Colonization	China	Gastronitestinal tract	Free living	No data	[30]
*Saccharomyces cerevisiae*	*B. mysticetus*	Colonization	Alaska	Skin	Free-living	No data	[29]
*Metschnikowia* spp.	*Balaenoptera musculus*	Colonization	California	Faeces	Free living	No data	[81]

## 3. The Significance of Non-*Candida* Yeasts in Cetacean Health and Disease

The diverse microbial communities within cetaceans, notably including non-*Candida* yeasts, are pivotal in shaping the health and disease dynamics of these marine mammals. This exploration transcends the immediate sphere of veterinary medicine and cetacean health, shedding light on broader ecological interactions and health implications. By delving into these intricate relationships, we not only contribute to cetacean conservation and well-being but also gain insights into the delicate balance of marine ecosystems. Such research underscores the symbiotic relationship between animal health and environmental integrity and, by extension, the well-being of human populations, aligning with a comprehensive approach to health that acknowledges the interconnectedness of all life forms and their shared environment.

As previously shown, several yeast species have been isolated from cetaceans, both from healthy and diseased individuals. While many of these yeasts, such as *Trichosporon* spp. and *Rhodotorula* spp., have been identified as environmental saprophytes, their detection in healthy cetaceans suggests potential commensal or even mutualistic relationships. For instance, they might play roles in nutrient absorption, immune system modulation, or protection against pathogenic microbes by outcompeting them or producing inhibitory compounds. Indeed, high-throughput sequencing in the gastrointestinal tract of East Asian finless porpoises revealed a high abundance of *Malassezia* spp. in the foregut, suggesting potential roles in digestion or maintaining gut homeostasis [30].

However, the separation between symbiosis and pathogenicity can be tenuous. While *Trichosporon* spp. have been isolated from healthy cetacean skin, they have also been implicated in cutaneous diseases, as noted in cases involving bottlenose dolphins [62]. Furthermore, *Coccidioides* spp., known to thrive in terrestrial environments, are responsible for respiratory infections in cetaceans, with fatal consequences in some cases [67,69]. Importantly, other fungal species such as *Cryptococcus* spp. and *Paracoccidioides* spp., as well as other dimorphic fungi, stand out for their potentially severe impact on cetacean well-being and might also be considerably misidentified and highly underdiagnosed, possibly due to the lack of access to these wild populations, among others [14,60].

Environmental disturbances, immune suppression due to pollution or climate alterations, or stress might facilitate these transitions from harmless colonization to disease. Understanding the triggers for this shift is critical for managing the health of both wild and captive cetaceans [6,82]. The diverse interactions of non-*Candida* yeasts with cetaceans might have profound implications for both conservation and veterinary medicine. Recognizing the potentially pathogenic role of some yeasts, especially in immunocompromised individuals, as well as the immune responses to these diseases and the host–pathogen relationship, emphasizes the importance of regular health assessments and monitoring, particularly in captive settings where animals might be exposed to various stressors or immunosuppressive therapies. Moreover, understanding cetacean mycobiome composition and function, which is still limited in the scientific literature beyond limited studies [30,81] could illuminate broader ecological dynamics, including how pollution, climate change, or human interventions affect marine ecosystems.

The health of cetaceans often reflects the health of their ecosystems [5]. From our perspective, disturbances leading to increased susceptibility to yeast infections, for instance, may signify broader ecological issues that need to be addressed. Alterations in the prevalence or pathogenicity of particular yeast species could serve as early warning signs of environmental changes, and preemptive fungal biomarkers could serve as valuable and useful indicators of individual, population, or ecosystem changes. For example, an increase in pathogenic fungi or a decrease in beneficial fungi might suggest that environmental stressors or contaminations affect the health of the marine ecosystem. Furthermore, for conservation efforts, understanding these dynamics becomes crucial. can be drawn.

## 4. Pathogenicity and Impact of Non-*Candida* Yeasts on Cetaceans

In recent years, scientific research has shifted focus from understanding the pathogenicity of *Candida* spp. to recognizing the potential risks posed by other yeast species, such as *Cryptococcus* or dimorphic fungi in other organisms, including cetaceans, especially considering the need for One Health approaches and the impacts of global climate change [14,83]. Although fungi are traditionally neglected pathogens, both in research and in the clinic [84], the current understanding of yeast pathogenicity in cetaceans but also in other animals and humans proves important for obtaining a multifaceted understanding of emerging infectious diseases, ecosystem dynamics, and the broader implications for global health and conservation strategies.

Non-*Candida* yeasts, such as *Malassezia*, *Cryptococcus*, and *Rhodotorula* species, are often commensals on a wide range of hosts. However, opportunistic pathogenicity, a common feature among these species, can be triggered under specific circumstances, such as immunosuppression or a breach of protective barriers. In terrestrial organisms, infections may manifest as skin disorders, fungemia, or even meningitis or pneumonia, particularly by *Cryptococcus* spp. These manifestations, although documented in various hosts, have not been comprehensively studied in cetaceans, and in many cases, such as the cutaneous isolations in lesioned skin in Alaskan bowhead whales [29], the proper definition of isolations as colonizing organisms, contamination, or pathogens is difficult.

Cetaceans, given their distinct physiology and wide range of ecological niches, present a unique setting for pathogen-host dynamics. The interplay between host, pathogen, and environmental factors dictates the positioning of any given pathogen on the spectrum of disease outcomes. Cetaceans, due to their unique respiratory adaptations suited to an exclusively marine environment, exhibit vulnerability to lower respiratory tract infections, such as pneumonia, induced by an array of pathogens [85]. The prevalence of these pathogens, including *Cryptococcus*, *Blastomyces*, and *Coccidioides*, is contingent upon their environmental epidemiology [25,65,69]. In this regard, changes in the ecological niches of both hosts and pathogens due to anthropogenic activities or climate change might also give rise to emerging infectious diseases [6]. In contrast to other mammals, cetaceans eschew conventional nasal filtering mechanisms in favor of blowholes, thereby facilitating the intake of substantial tidal volumes into the respiratory airway via profound inspiration prior to diving [14]. Such a physiological adaptation renders these species particularly prone to acquiring cryptococcosis, among others, in regions with an elevated incidence of infective propagules. Consequently, the invasion of the respiratory tract by these pathogens may precipitate disseminated infections, a risk augmented when other factors, such as additional environmental stressors, compromise host immunity.

Furthermore, the thick blubber layer, while essential for thermoregulation, may also become a reservoir for yeast colonization, especially if wounds or breaches are present. In addition to *Cryptococcus* spp., *Trichosporon* spp., and *Candida* spp. [29], lipid-dependent organisms such as *Malassezia*, which are often implicated in skin conditions in humans and other mammals, might exploit such breaches in cetaceans. Yet, the true magnitude and nature of such infections are largely speculative.

*Cryptococcus gattii* is another example of how the distinct anatomophysiological features of these animals model the pathogenicity of fungal infections. Primarily a pathogen of terrestrial origin, *C. gattii* has been isolated from marine environments, highlighting the yeast’s adaptability [17,24]. While *C. gattii* has demonstrated neurotropism in terrestrial mammals, including humans, leading to severe CNS infections, the lack of a cribriform plate in cetaceans decreases the frequency of these infections, although cases of cryptococcosis with CNS involvement in cetaceans have been demonstrated [19].

The gastrointestinal tract of cetaceans, a vital interface for nutrient absorption and host–microbe interactions, might be susceptible to non-*Candida* yeast colonization or invasion. In terrestrial mammals, the overgrowth of certain yeasts, such as *Rhodotorula* spp., has been linked to gastrointestinal disturbances. By analogy, it is conceivable that similar overgrowths in cetaceans, especially when associated with environmental stressors or dietary shifts, might compromise their gastrointestinal health. Indeed, the gastrointestinal mycobiome has been largely underexplored, with only two high-throughput sequencing analyses carried out until now [30,81].

These studies revealed that *Malassezia* spp. And *Metschnikowia* spp. Dominate the gut mycobiomes of East Asian finless porpoises and blue whales, respectively. However, the limited sample size and genus-level resolution emphasize the need for further research to better understand the complex microbiome of cetaceans, including their interactions with pathogens and commensals.

The cumulative impacts of non-*Candida* yeast infections on the overall health of cetaceans might range from negligible to severe, potentially exacerbating other health concerns or decreasing their fitness. Such impacts may further influence cetacean behaviors, reproductive success, or even survival rates [6].

## 5. Environmental Influence and Health Implications of Non-*Candida* Yeasts in Cetaceans

Marine ecosystems are highly sensitive to environmental changes. They are currently facing a wide range of anthropogenic perturbations, such as climate change or pollution, with distinct responses at different trophic levels that may disrupt ecological interactions and thereby threaten marine ecosystem function [86,87]. This influence significantly alters microbial community structures. Nonetheless, predicting the implications of these changes on ecosystem functionality, especially in the long term, remains challenging, especially if the stressor persists or intensifies [88]. Despite their pivotal role in ecosystem dynamics, microorganisms, especially yeasts, have been underexamined, largely due to challenges in analyzing their vast diversity and the traditional regard for fungal microorganisms in scientific research. However, the fungal community is a valuable indicator of anthropogenic activities in aquatic ecosystems [89]. Microbial communities react to higher temperatures, nutrients, and chemical pollutants with increasing cell counts. Concurrently, shifts in community structure heighten diversity, and pronounced temporal fluctuations occur. Such transformations, indicative of environmental changes from human-induced stress, can impact yeast community functions and may pose health risks both to the environment and cetaceans, as well as to humans, as indicated by the proliferation and emergence of pathogens and increased antifungal resistance [88,90,91].

Marine filamentous fungi are primarily found in coastal areas such as mangroves and driftwood, while yeasts are widespread in both open seas and deep-sea domains [90]. The ubiquitous presence of these fungi in marine ecosystems facilitates their colonization of marine animals and promotes interactions across diverse marine habitats. In fact, recent molecular studies have led to the identification of new marine fungal species belonging to genera previously described in cetaceans such as *Cryptococcus*, *Candida*, *Rhodotorula*, and even *Malassezia* [90,92,93].

Increasing ocean temperatures can modify the yeast microbiota by favoring those species that thrive in warmer waters, potentially leading not only to an imbalance but also to the selection of more virulent species and strains [94]. This, in turn, might pave the way for opportunistic yeast pathogens that might be innocuous under regular circumstances. For instance, temperature fluctuations could increase the proliferation rates of certain yeast strains, which might not necessarily be benign to their cetacean hosts. Furthermore, the epidemiology of certain yeast species has changed as a consequence of global change [95].

On the one hand, paracoccidioidomycosis and cryptococcosis are highly impacted by global warming. Climatic alterations have been intricately linked to shifts in paracoccidioidomycosis distribution and prevalence [96]. Increased temperatures, humidity, and strong El Niño events as a consequence of climate change have been linked to clusters of paracoccidioidomycosis cases [97]. Previously, *C. gattii* was found to be confined to tropical regions, but recent findings have reported its presence in the Mediterranean, the USA, and Canada, with this northward movement being alarming [18,98]. Its geographic spread may be attributed to various factors, including global trade and global warming [99,100], and has translated to severe outbreaks in cetaceans [16,17,18,21,22,24]. Additionally, thermal adaptation has been associated with increased virulence [94].

On the other hand, environmental shifts could influence the rising incidence of dimorphic fungi in marine mammals. *Coccidioides* spp. have been proven to thrive in marine environments and infect marine mammals, including cetaceans [53,69], and unusual weather patterns have been linked to a significant increase in cases in recent decades [101]. The predicted surge in dust storms and the spread of arid environments, together with soil disruption and periods of changes in precipitation [95], could potentially double endemic areas and transport a large number of infective propagules into the marine environment, becoming a threat to cetaceans. These environmental disturbances apply to other dimorphic fungi, such as *Histoplasma* spp. [102] and *Blastomyces* spp. [103]. Additionally, in the case of *Histoplasma* spp., climate-influenced behavioral shifts in birds and bats are anticipated to affect the transmission dynamics of histoplasmosis [104]. Specific *H. capsulatum* strains, exhibiting enhanced virulence under conditions of elevated temperature or augmented light exposure, suggest potential evolutionary pressures. Given the predicted increases in temperature and UV exposure, there is a plausible risk for the emergence of increasingly pathogenic strains affecting less common hosts such as cetaceans [105,106].

Pollution has multiple effects on marine biota. Industrial and agricultural runoff introduces numerous chemicals into the marine ecosystems—from heavy metals to persistent organic pollutants and antifungal agents [107]. In addition to their direct toxic effects, these xenobiotics can also modulate the microbial community structure, including that of yeasts [107]. Certain yeasts exhibit increased resistance to pollutants, thrive in compromised environments, and potentially displace less-resistant commensal species. Many pathogenic yeast species affecting both humans and cetaceans, such as *Candida*, *Rhodotorula*, and *Trichosporon*, have been strongly associated with polluted marine environments [108,109]. This higher resistance to pollutants as well as antifungal agents, which can proliferate in compromised environments, might potentially displace less-resistant, commensal species [110,111]. This change in yeast dynamics could hypothetically have broader implications. As these resistant yeasts become more prevalent, they might alter the interactions within the marine microbiome and with their macrobiotic hosts, such as cetaceans, as in humans [112]. Moreover, the displacement of less-resistant commensal yeast species might perturb the health equilibrium of marine hosts, leading to increased susceptibility to diseases or other health anomalies. Furthermore, this equilibrium is further affected by the negative health impacts of pollutants in the host.

Although there is limited information on the cetacean microbiome and virtually no scientific evidence regarding the mycobiome of these organisms using comprehensive genome sequencing techniques, the gastrointestinal tract of cetaceans harbors a rich microbial consortium, with yeasts contributing to this diversity [11,30,81]. Dysbiosis could lead to alterations in proper digestion and nutrient uptake, rendering cetaceans vulnerable to nutritional shortfalls and gastrointestinal ailments. Moreover, environmental factors also have a direct influence on animals’ susceptibility and influence the onset and severity of outbreaks related to other emerging infections such as morbillivirus, paracoccidioidomycosis, toxoplasmosis, poxvirus-linked tattoo skin disease, and multifactorial infections in harbor porpoises. Coastal and estuarine cetaceans are supposed to face greater threats than their pelagic counterparts due to habitats being profoundly impacted by human-induced factors, including chemical and biological pollution, among others such as direct anthropogenic contact or climate disturbances [6]. Notably, pollution, notably through endocrine-disrupting chemicals [113] and immunotoxicity [114] can notably increase susceptibility not only to pathogenic fungal species but also to opportunistic yeasts. As has been described for lung cryptococcosis, histoplasmosis, and coccidiomycosis [17,25,27,69], yeasts can infiltrate the respiratory tract, potentially inducing pneumonia and developing systemic disease, especially in immunosuppressed cetaceans already weakened by external stressors. Likewise, fungal-associated skin infections might become increasingly frequent when natural barriers are compromised in pollutant-rich waters.

Overall, we conclude that environmental changes may influence the fungal populations, increase the pathogenicity of these microorganisms, or decrease the resilience of their hosts. Many of these ecological parameters do not operate in isolation. The constellation of rising temperatures, increased pollution, ocean acidification, and other anthropogenic influences creates a multifaceted and interconnected stress environment for marine organisms that can have synergistic negative effects, making predictions about individual stressor impacts challenging yet crucial. A comprehensive approach, merging marine biology, mycology, and environmental science, is crucial for elucidating the intricate interactions that occurred in the Anthropocene.

## 6. Knowledge Gaps and Future Directions for Non-*Candida* Yeasts in Cetacean Research

Advancements in next-generation sequencing (NGS) technology have deepened our understanding of human and animal microbiomes, facilitating the identification and description of previously unculturable microorganisms while also aiding in predicting their physiological and ecological roles [115]. Owing to the use of new molecular techniques such as 16S rRNA gene sequencing, shotgun metagenomic sequencing, and RNA sequencing, in recent years, the study of the bacterial microbiome in cetaceans has garnered significant attention. However, the fungal microbiome remains notably under investigated. Notably, there is an absence of comprehensive research focusing on the cetacean mycobiome in both wild and captive animals. Most evidence about fungal populations as well as infections in cetaceans is based on isolated case reports. While they provide invaluable insights, many studies have technical and microbiological limitations, making it challenging to generalize findings across broader cetacean populations. This knowledge gap limits our understanding of the diverse microbial communities and their ecological and pathophysiological roles within these marine mammals. Furthermore, the lack of data on antifungal susceptibility in yeast isolates from cetaceans is significant, especially considering the upcoming increase in antimicrobial resistance, the emerging fungal infections worldwide, and the direct relationship between antifungal susceptibility and anthropogenic environmental changes. This is particularly concerning given the potential role of treatment and conservation efforts, as well as its One Health implications that may reach public health.

These gaps offer potential avenues for future research. Undertaking systematic microbiological studies using culture-dependent and DNA-based techniques can provide insights into the diversity of yeasts in cetaceans, offering a foundational understanding of their mycobiome composition. This investigation of the equilibrium between commensal and pathogenic species and the symbiotic interactions between cetaceans and their yeasts can enhance our understanding of the benefits and potential drawbacks of these microbial communities. The impact of environmental factors on the cetacean mycobiome also deserves in-depth exploration, as does determining the pathogenicity of non-*Candida* yeasts, both for providing insights into potential disease outbreaks, informing conservation efforts, and predicting larger-scale ecosystem changes. To conclude, while the importance of understanding the mycobiome in cetaceans is undeniable, the field remains highly unexplored. Addressing these gaps will not only improve our knowledge of cetacean health and ecology but also have an impact on human and environmental health and inform conservation strategies from the so-needed One Health approach. As marine mammals face rising environmental challenges, a detailed understanding of all facets of their biology, including their mycobiome, becomes more crucial than ever.

## Data Availability

Not applicable.
